# Gut Microbiome as a Potential Marker of Hematologic Recovery Following Induction Therapy in Acute Myeloid Leukemia Patients

**DOI:** 10.1002/cam4.70501

**Published:** 2025-01-27

**Authors:** Valentina Salvestrini, Gabriele Conti, Federica D'Amico, Gianluca Cristiano, Marco Candela, Michele Cavo, Silvia Turroni, Antonio Curti

**Affiliations:** ^1^ Istituto di Ematologia "Seràgnoli" IRCCS Azienda Ospedaliero‐Universitaria di Bologna Bologna Italy; ^2^ Human Microbiomics Unit, Department of Medical and Surgical Sciences University of Bologna Bologna Italy; ^3^ Unit of Microbiome Science and Biotechnology, Department of Pharmacy and Biotechnology University of Bologna Bologna Italy; ^4^ Oncology and Haematology Research Area, Department of Medical and Surgical Sciences University of Bologna Bologna Italy

**Keywords:** acute myeloid leukemia, gut microbiome, hematological recovery, induction therapy

## Abstract

**Background:**

The management of acute myeloid leukemia (AML) is hindered by treatment‐related toxicities and complications, particularly cytopenia, which remains a leading cause of mortality. Given the pivotal role of the gut microbiota (GM) in hemopoiesis and immune regulation, we investigated its impact on hematologic recovery during AML induction therapy.

**Methods:**

We profiled the GM of 27 newly diagnosed adult AML patients using 16S rRNA amplicon sequencing and correlated it with key clinical parameters before and after induction therapy.

**Results:**

Our investigation revealed intriguing associations between the GM composition and crucial recovery indicators, including platelet, lymphocyte, and neutrophil counts, and identified early GM signatures predictive of improved hematologic recovery. Remarkably, patients demonstrating superior recovery had higher alpha diversity and enrichment in health‐associated taxa belonging to the genera *Faecalibacterium*, *Ruminococcus*, *Blautia*, and *Butyricimonas* at diagnosis.

**Conclusions:**

Despite certain study limitations, our findings suggest that evaluating GM features could serve as a potential marker for hematologic recovery. This preliminary work opens avenues for personalized risk assessment and interventions, possibly involving GM modulation tools, to optimize recovery in AML patients undergoing induction therapy and potentially enhancing overall outcomes in individuals with hematologic diseases.

## 1 Introduction

Acute myeloid leukemia (AML) is an aggressive heterogeneous disease. Despite recent advances, the 5‐year overall survival (OS) of AML patients is still largely unsatisfactory, reaching only 30% and dropping to 5%–10% in the elderly [1, 2]. Treatment‐related toxicities and complications of cytopenias, such as bleeding and infections, are the major limiting factors in leukemia treatment and the leading cause of death in this disease [3].

The gut microbiota (GM), i.e., the largest human‐associated assemblage of microorganisms residing in the gastrointestinal tract, is a key orchestrator of host health [4, 5]. Among others, GM is known to be directly involved in modulating the immune system and regulating hemopoiesis, thereby affecting the host response to inflammation and infection [4, 5, 6, 7]. It is therefore not surprising that an altered (i.e., dysbiotic) GM profile has been associated with increased susceptibility to infection [8, 9, 10] and a plethora of inflammation‐related disorders (from intestinal to metabolic, hepatic, respiratory, cardiovascular, neurologic, and oncologic) [11, 12]. Not least, GM has also been shown to affect the outcome of several treatments, including anti‐cancer immunotherapy [5, 13, 14]. Regarding hematologic malignancies, several studies have consistently described profound GM dysbiosis in both adult and pediatric patients, including those with AML [6, 10, 15, 16, 17, 18]. Furthermore, GM diversity and specific compositional signatures have been shown to be predictive of infectious risk following induction therapy [6, 10, 19] and of several complications associated with allogeneic stem cell transplantation (allo‐HSCT), such as graft‐versus‐host disease (GvHD) and mortality, in leukemia, lymphoma, and myeloma patients [6, 20, 21]. However, as far as we know, to date, no studies have explored the relationship between GM and hematologic recovery after induction therapy.

To fill this gap, here, we profiled the GM of AML patients at diagnosis through 16S rRNA amplicon sequencing and correlated it with a number of key clinical parameters, including hematologic recovery after therapy initiation.

## 2 Materials and Methods

### 2.1 Patients and Fecal Sampling

Stool samples from 27 adult AML patients at diagnosis were collected 1 to 2 days before the initiation of induction therapy at IRCCS University Hospital of Bologna (Italy). The study was approved by the Independent Ethics Committee of the Area Vasta Emilia Centro (approval code: 94/2016/O/Tess), and patients gave written informed consent.

### 2.2 16S rRNA Gene Amplification and Illumina Sequencing

Microbial DNA was extracted from fecal samples using the repeated bead‐beating plus column method, as previously described [22]. In brief, feces (250 mg) were suspended in tubes containing four 3 mm glass beads, 0.5 g of 0.1 mm zirconia beads, and 1 mL of lysis buffer [500 mM NaCl, 50 mM Tris–HCl, 50 mM EDTA, 4% (w/v) SDS] and mechanically homogenized thrice in a FastPrep instrument (MP Biomedicals, Irvine, CA, USA) at five movements/s with a 1 min interval every 5 min. Subsequently, the samples were treated at 95°C for 15 min, and a centrifugation step was performed to settle beads and stool particles. After 10 min incubation with 10 M ammonium acetate, nucleic acids were precipitated by adding one volume of isopropanol. Pellets were washed in 70% ethanol and resuspended in 10 mM Tris–HCl, 1 mM EDTA pH 8.0 buffer. After treatment with DNase‐free RNase (10 mg/mL) at 37°C for 15 min, samples were subjected to protein removal and on‐column DNA purification using the DNeasy Blood and Tissue Kit (QIAGEN, Hilden, Germany).

For amplification of the V3–V4 region of the 16S rRNA gene, the primers 341F and 785R with added Illumina adapter overhang sequences were used as previously described [22]. PCR amplification was performed using the KAPA HiFi Hot Start Ready Mix (Roche, Basel, Switzerland) following the Illumina protocol “16S Metagenomic Sequencing Library Preparation” (Illumina, San Diego, CA, USA). A magnetic bead‐based clean‐up system (Agencourt AMPure XP; Beckman Coulter, Brea, CA, USA) was used to purify the amplicons, and limited‐cycle PCR using Nextera technology was performed to prepare the indexed libraries. After further purification and pooling to an equimolar concentration (4 nM), the pool was denatured with 0.2 N NaOH and diluted to 4.5 pM with a 20% PhiX control before paired‐end sequencing (2 × 250 bp) on an Illumina MiSeq platform. Sequence reads were deposited in the National Center for Biotechnology Information Sequence Read Archive (NCBI SRA, BioProject: PRJNA1139414).

### 2.3 Bioinformatics and Statistics

Raw sequences were analyzed using a combined PANDASeq [23] 23 and QIIME 2 [24] pipeline. After length and quality filtering, reads were clustered into amplicon sequence variants (ASVs) using DADA2 [25]. Taxonomy was assigned using the VSEARCH algorithm [26] on the Greengenes database, and chimeras were discarded during the process. Alpha and beta diversity were assessed using the q2 diversity plugin. Alpha diversity was evaluated using the Shannon index, observed species, and Pielou's evenness, while weighted and unweighted UniFrac distances were used to construct Principal Coordinates Analysis (PCoA) graphs. We primarily reported the Shannon index in the main figures, as it provides a balanced measure by incorporating both richness and evenness, especially when results across different metrics were comparable.

All statistical analyses were performed using R software (v. 4.4.0). The Wilcoxon test was used as the primary method to assess differences in GM composition and alpha diversity across binary patient groups, ensuring consistency and suitability for our sample size and the not normally distributed data tested. Images were done using ggplot2 [27] and MicrobAIDeR [28] R packages. PCoA plots were generated using the vegan (v 2.6‐2, //CRAN.R‐project.org/package=vegan) and Made4 [29] packages, and data separation was tested by a permutation test with pseudo‐F ratio (function Adonis in vegan). To represent taxa, relative abundance differences of reported GM taxa, boxplots based on relative abundance values, or barplots based on log fold change (LFC) were used. The LFC was calculated as: LFC = $\log\left(\frac{c_{1}\cdot {\text{Median}}_{1}+\left(1-c_{1}\right)\cdot {\text{Mean}}_{1}+0.001}{c_{2}\cdot {\text{Median}}_{2}+\left(1-c_{2}\right)\cdot {\text{Mean}}_{2}+0.001}\right)$ where c1=c2=0.7. Bacterial taxa were filtered by a prevalence greater than 0.2 in at least two samples, resulting in 34 families and 42 genera being included for the analysis. Spearman and point‐biserial correlation tests were used to evaluate associations between relative taxon abundances and continuous (i.e., hematologic) or dichotomous (presence of mutations, antibiotic administration, induction regimen, and OS) patient metadata, respectively. Only statistically significant correlations (*p* ≤ 0.05) were considered. *p* values were corrected for multiple comparisons using the Benjamini–Hochberg method. A false discovery rate (FDR) ≤ 0.05 was considered statistically significant. FDR ≤ 0.1 was considered a trend.

## 3 Results

### 3.1 Study Cohort Description

Twenty‐seven patients with new‐onset AML were enrolled at the Hematology Unit of IRCCS Azienda Ospedaliero‐Universitaria of Bologna, Italy. Patient characteristics are shown in Table 1. The median age was 56.5 years (range, 23–74); 10 patients were female and 17 male; 8 patients had hyperleukocytosis at onset defined as white blood cell (WBC) count > 30,000/mm^3^. Regarding the cytogenetic and molecular data of the disease at onset, according to the European LeukemiaNet (ELN) 2017 prognostic classification [2] (due to the lack of next‐generation sequencing data for all patients at diagnosis, we could not classify patients according to the updated guidelines), 5 patients were at low ENL risk, 15 at intermediate risk, and 7 at high risk (6 featured by complex karyotype). When molecular biology was evaluable, 8 patients had *FLT3* (internal tandem duplication, *ITD* or tyrosine kinase domain, *TKD*) mutations, 8 had *NPM1* mutations, 4 had IDH1/2 mutations and 3 had *TP53* mutations. Twenty‐six patients were given induction therapy (one died before induction), of whom 22 received intensive chemotherapy, including 14 patients on fludarabine‐based regimens (fludarabine, cytarabine, and idarubicin Days 5 and 3, FLAI5 and FLAI3) and 8 patients on “3 + 7”‐based regimens (7 with the addition of midostaurin as FLT3‐positive), whereas 4 were treated with a 10‐day of hypomethylating agent decitabine. Of the 25 patients evaluable for response to therapy, 17 achieved a complete response (CR) to first‐line therapy (9 of which also achieved Minimal Residual Disease (MRD) negativization), while 8 were therapy‐refractory. As for inflammatory markers, 19 out of 27 patients had high ferritin (> 400 ng/mL) and elevated C‐reactive protein (CRP) levels with a 0.5 mg/dL cut‐off. Regarding infectious issues, 9 out of 27 patients had received empirical broad‐spectrum antibiotic therapy within 30 days prior to stool collection and induction therapy; 13 patients were on neutropenia prophylaxis within the same 30‐day period (see Table S1 for details on the specific drugs used). During post‐therapy neutropenia, all patients treated with intensive chemotherapy experienced one or multiple fever episodes that were treated and resolved with broad‐spectrum antibiotic therapy; microbial agents were isolated in blood samples from 11 patients, including *Escherichia coli* ESBL, *Pseudomonas aeruginosa* , *Klebsiella pneumoniae* KPC, *Enterococcus faecium* , and *Stenotrophomonas maltophilia* . Median relapse‐free survival (RFS) and OS in the whole cohort were 10 months (range, 1–46) and 20 months (range, 2–46), respectively.

**TABLE 1:** Patient characteristics.

#Pts	Age	Sex	WBC, cells/μL	Karyotype	FLT3	IDH1/2	TP53	NPM1	Risk^a^
1	60	F	3400	Normal	wt	wt	wt	mut	Low
2	43	M	62,400	Complex	ITD+	wt	n.a.	wt	High
3	64	M	2500	Normal	wt	wt	wt	wt	Intermediate
4	70	M	11,400	+(8)	wt	n.a.	n.a.	wt	Intermediate
5	68	M	57,300	Normal	TKD+	wt	wt	wt	Intermediate
6	68	F	11,000	Complex	wt	n.a.	n.a.	wt	High
7	67	M	2400	Normal	wt	wt	wt	wt	Intermediate
8	71	M	13,400	− (17)	wt	wt	n.a.	wt	High
9	72	F	3600	Normal	ITD+	wt	wt	mut	Intermediate
10	47	F	2300	Normal	ITD+	wt	wt	mut	Intermediate
11	26	F	6000	Normal	wt	wt	wt	mut	Low
12	49	M	n.a.	Normal	wt	wt	wt	wt	Intermediate
13	51	M	2000	Normal	wt	IDH1+	n.a.	wt	Intermediate
14	74	M	1600	Complex	wt	wt	mut	wt	High
15	47	F	4610	Normal	ITD+	wt	wt	mut	Intermediate
16	44	F	35,000	Normal	wt	IDH1+	wt	mut	Low
17	45	M	10,300	Complex	wt	wt	wt	wt	High
18	67	F	45,600	Normal	wt	IDH1+	wt	mut	Low
19	59	M	179,210	del (9)	ITD+	n.a.	n.a.	wt	Intermediate
20	66	M	61,800	+(12)	TKD+	n.a.	n.a.	wt	Intermediate
21	59	M	2200	Normal	TKD+	wt	wt	wt	Intermediate
22	52	M	88,630	Complex	wt	wt	wt	wt	High
23	58	F	120,000	Normal	wt	IDH1+	wt	mut	Low
24	72	M	2200	n.a.	n.a.	n.a.	mut	n.a.	Intermediate
25	58	M	n.a.	n.a.	wt	wt	wt	wt	Intermediate
26	23	M	11,400	Normal	wt	wt	wt	wt	Intermediate
27	46	F	2700	Complex	wt	wt	mut	wt	High

Abbreviations: n.a., not available; WBC, white blood cell.

^a^ Following ELN2017.

### 3.2 Hematologic Recovery After Induction Therapy

At different time points after induction therapy (15, 21, 28 days, and before consolidation therapy), absolute lymphocyte count (ALC), absolute neutrophil count (ANC), and platelet count (PLT) were obtained. Hematologic recovery was assessed using the following cutoff values: Full recovery (FR) for PLT ≥ 50,000/mm^3^ and ALC and ANC ≥ 500/mm^3^, no recovery (NR) for PLT < 50,000/mm^3^ and ALC and ANC < 500/mm^3^. According to the literature [30], ALC has a role in predicting progression‐free survival (PFS) and OS when assessed after induction chemotherapy in adult AML patients. Specifically, ALC recovery ≥ 500/mm^3^ has been shown to confer a survival advantage. Due to prolonged neutropenia, four patients were administered subcutaneous granulocyte colony‐stimulating factor until ANC recovery. Twenty‐two out of 27 patients were evaluable for hematologic recovery (Figure S1), and all of them received intensive chemotherapy. Of note, the ALC value of 500/mm^3^ was reached only since Day 28 in 12 patients, since Day 21 in 8 patients and, remarkably, since Day 15 in 5 patients. This early ALC recovery is known to be the strongest prognostic factor according to the literature. Indeed, these five patients achieved CR and proceeded to HSCT consolidation. We could not directly correlate ALC recovery as a clinical biomarker to infection occurrence; nevertheless, in our cohort, patients who had achieved ALC recovery had not experienced prolonged fever episodes nor septic status and did not have microbiological isolates. As expected, the same considerations could be made for patients who rapidly recovered ANC. Regarding PLT recovery, on Day 21, 10 out of 22 patients had already reached the 50,000/mm^3^ cutoff without ANC recovery; all of them achieved CR after induction therapy, and most of them proved to be MRD‐negative after subsequent courses. Nine of them were alive and in CR at the time of writing, and only two achieved consensual ALC recovery at all time points considered. It is noteworthy that patients who achieved platelet recovery did not receive any platelet support in the days prior to evaluation, thereby confirming that their recovery was not influenced by transfusions.

### 3.3 Gut Microbiota Profile of AML Patients at Diagnosis Is Not Associated With Cytogenetic and Molecular Data of the Disease at Onset and Response to Therapy

Fecal samples were collected at diagnosis for the 27 enrolled AML patients and subjected to 16S rRNA amplicon sequencing, yielding a total of 5,371,200 reads (mean ± SD, 198,933 ± 45,126) binned into 3450 ASVs.

**FIGURE 1:**
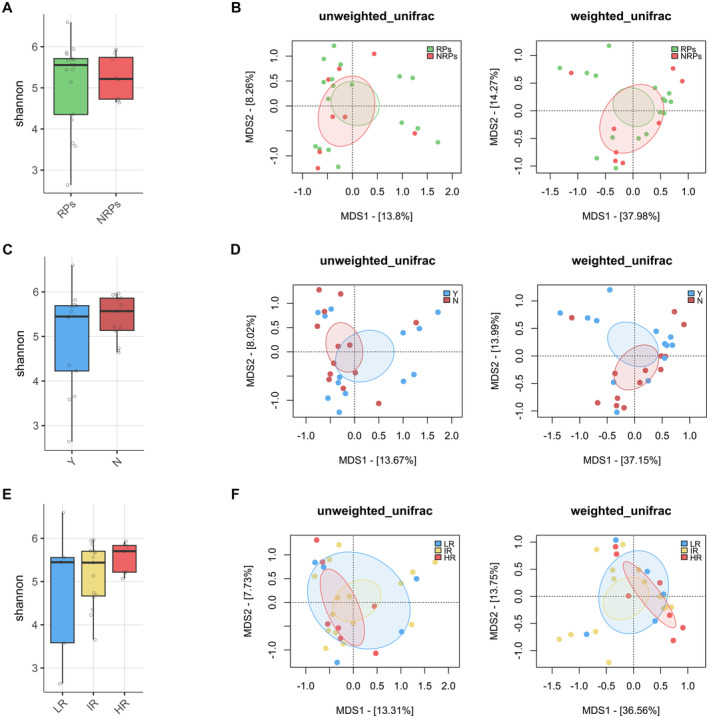
Alpha and beta diversity of the gut microbiota of AML patients at diagnosis stratified by response to therapy, OS, and ELN risk. Alpha diversity was assessed using the Shannon index, which accounts for both richness and evenness of microbial communities, while beta diversity was evaluated using unweighted and weighted UniFrac distances to capture compositional dissimilarity based on phylogenetic relationships. For alpha diversity comparisons, the Wilcoxon rank‐sum test with FDR *p*‐value correction was applied, and for beta diversity, PERMANOVA test was used to test for differences between groups. Ellipses include 95% confidence intervals based on the standard error of the weighted average of sample coordinates. No significant separation was found (PERMANOVA, *p* > 0.1). (A, B) Associations of alpha and beta diversity with response to therapy (RPs = responder, NRPs = nonresponder, *n* = 25). (C, D) Associations of alpha and beta diversity with OS (*n* = 26), patients stratified by who survive (Y) or did not survive (N). (E, F) Evaluation of alpha and beta diversity in patients stratified by ELN risk (LR = low risk, IR = intermediate risk, HR = high risk, *n* = 27).

**FIGURE 2:**
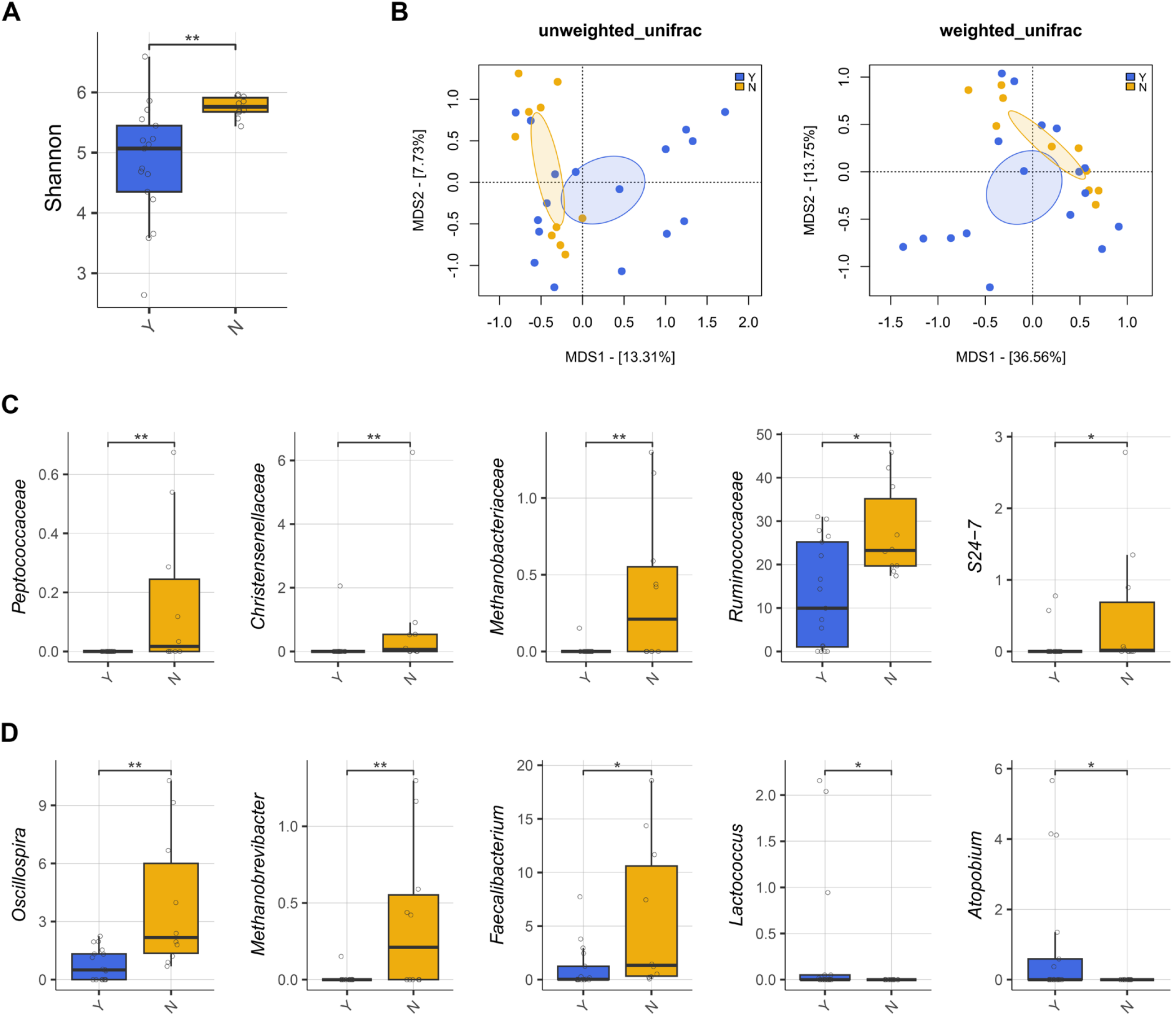
The gut microbiota of AML patients at diagnosis varies by antibiotic treatment. (A) Boxplot showing the distribution of alpha diversity, estimated with the Shannon index, in the gut microbiota of patients receiving or not receiving antibiotics (Y vs. N, *n* = 27) prior to induction therapy. Wilcoxon's test, ** for FDR‐corrected *p*‐value < 0.01. (B) PCoA plots based on unweighted and weighted UniFrac distances between the two patient groups. Ellipses include 95% confidence area based on the standard error of the weighted average of sample coordinates. A significant separation was found with the unweighted UniFrac distances (PERMANOVA, *p* = 0.034). Boxplots showing the relative abundance distribution of families (C) and genera (D) differentially represented between the two patient groups. Wilcoxon's test with FDR‐corrected *p*‐value, *p ≤ 0.05, **p < 0.01.

The GM profile was first analyzed in relation to patient metadata at diagnosis. Potential confounders were tested, and alpha and beta diversity analyses showed no significant differences (*p* < 0.05) in patients stratified by age, sex, or hyperleukocytosis, defined by WBC > 30,000/mm^3^ (Figure S2). Key clinical parameters were then tested to assess GM diversity differences in relation to clinical response (RPs and NRPs), overall survival (OS), and ELN risk (Figure 1). No significant separation was found in beta PCoAs also for the presence of *FLT3*, *NPM1*, IDH1/2, *TP53* mutations, and karyotype alterations (PERMANOVA, *p* > 0.1) (Figure S3). In contrast, patients receiving antibiotics prior to sample collection exhibited reduced GM diversity compared to untreated patients (Wilcoxon's test with FDR correction, *p* = 0.001) and segregated differently in the unweighted UniFrac‐based PCoA (PERMANOVA, *p* = 0.034) (Figure 2A,B). From the taxonomic standpoint, patients on antibiotic therapy prior to sample collection showed reduced levels of *Peptococcaceae, Christensenellaceae*, *Methanobacteriaceae* (and its genus *Methanobrevibacter*), *S24‐7* (also known as *Muribaculaceae*), and *Ruminococcaceae* (including *Oscillospira* and *Faecalibacterium*) while increased levels of *Lactococcus* and *Atopobium* compared to untreated patients (Wilcoxon's test with FDR correction, *p* < 0.05) (Figure 2C,D).

We then looked for associations between the GM profile, response to therapy, and the occurrence of post‐therapy infections. No significant differences in alpha or beta diversity were observed associated to therapy response or OS at diagnosis, whether patients were analyzed collectively (Figure 1) or stratified by treatment modality, specifically focusing on those who received chemotherapy (Figure S4), as only four patients were treated with hypomethylating agents. Regarding infections (Figure S5), a trend toward segregation was observed in the weighted UniFrac‐based PCoA between patients who underwent infection compared to non‐infected patients (PERMANOVA, *p* = 0.089). In particular, patients who developed an infection after therapy had increased levels of *Lactococcus* and decreased levels of *Dorea* and *Anaerotruncus* (Wilcoxon's test with FDR correction, *p* < 0.05).

### 3.4 Gut Microbiota Profile of AML Patients at Diagnosis Is Associated With Hematologic Recovery After Induction Therapy

**FIGURE 3:**
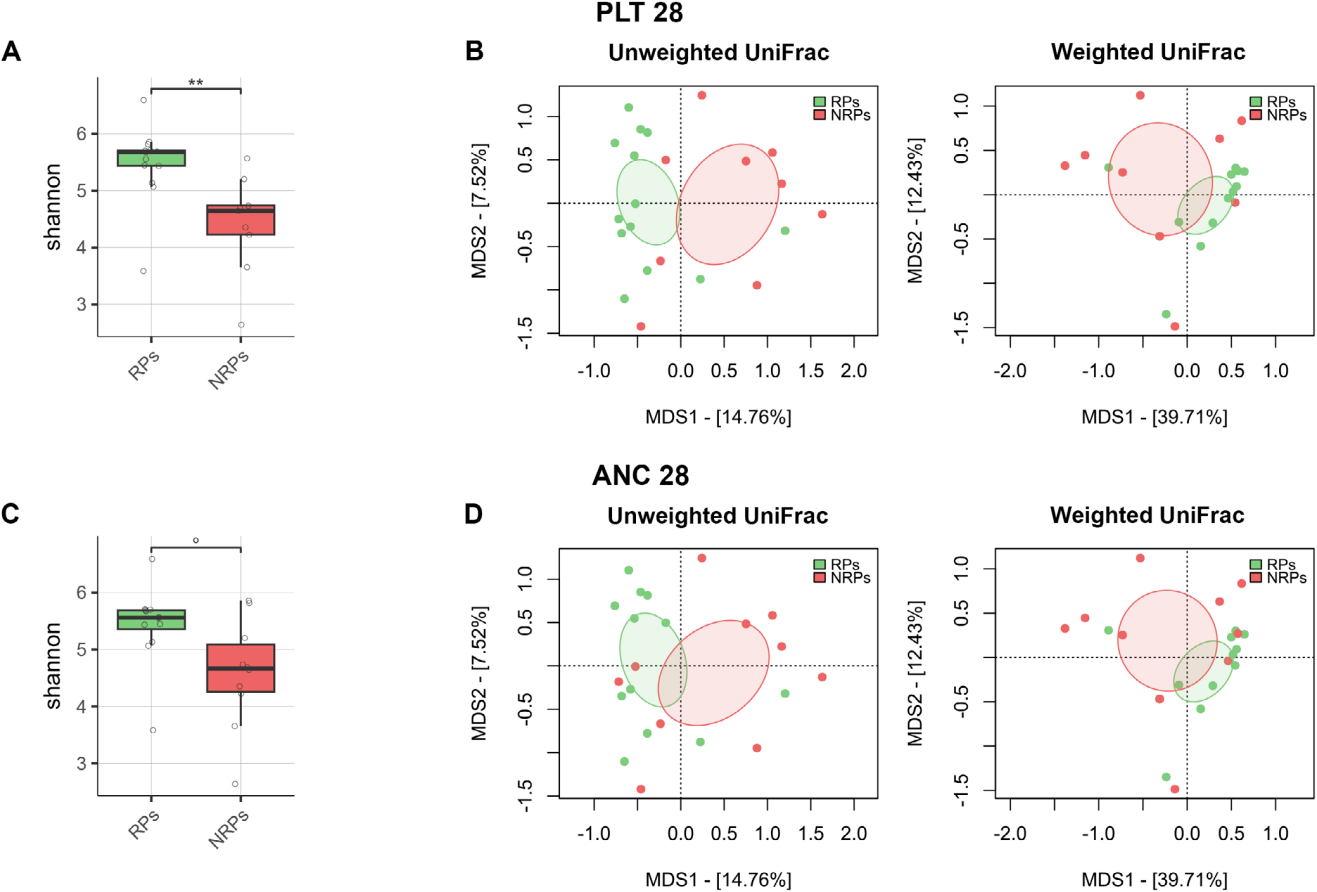
Alpha and beta diversity of the gut microbiota of AML patients at diagnosis stratified by hematologic recovery. Boxplots showing the distribution of alpha diversity, estimated with the Shannon index, in the gut microbiota of patients who recovered platelet count (PLT 28, A) or absolute neutrophil count (ANC 28, C) (RPs) and those who did not (NRPs) on Day 28 after induction therapy. Wilcoxon's test with FDR‐corrected *p*‐value, ** for *p* < 0.01, and° for *p* = 0.05. PCoA plots based on unweighted and weighted UniFrac distances between gut microbiota profiles of RPs and NRPs for PLT 28 (B) and ANC 28 (D). Ellipses include 95% confidence area based on the standard error of the weighted average of sample coordinates. For PLT 28, a significant separation was found in both unweighted and weighted UniFrac‐based PCoA plots (PERMANOVA, *p* ≤ 0.02); for ANC 28, a significant separation was found in the unweighted UniFrac‐based PCoA plot (*p* = 0.04), while only a trend was found with the weighted UniFrac metric (*p* = 0.1). *N* = 22.

**FIGURE 4:**
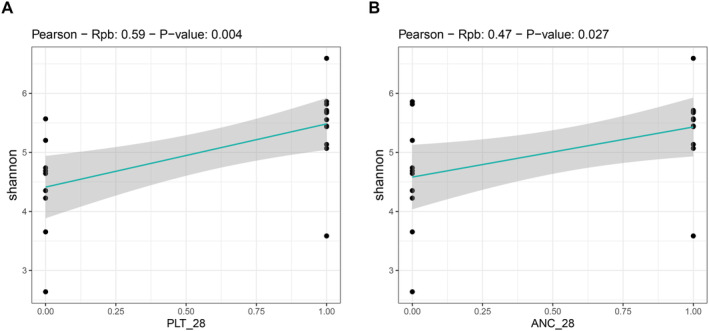
Correlation between the alpha diversity of the gut microbiota of AML patients at diagnosis and hematologic recovery after induction therapy. Scatterplots showing the point‐biserial correlations (Rpb) between alpha diversity, estimated with the Shannon index, and platelet count (PLT, A) or absolute neutrophil count (ANC, B) on Day 28 after induction therapy, expressed as a dichotomous variable with 0 for nonrecovery and 1 for recovery. Rpb is equivalent to Pearson's correlation coefficient (Phi) when comparing continuous and dichotomous variables. *N* = 22.

**FIGURE 5:**
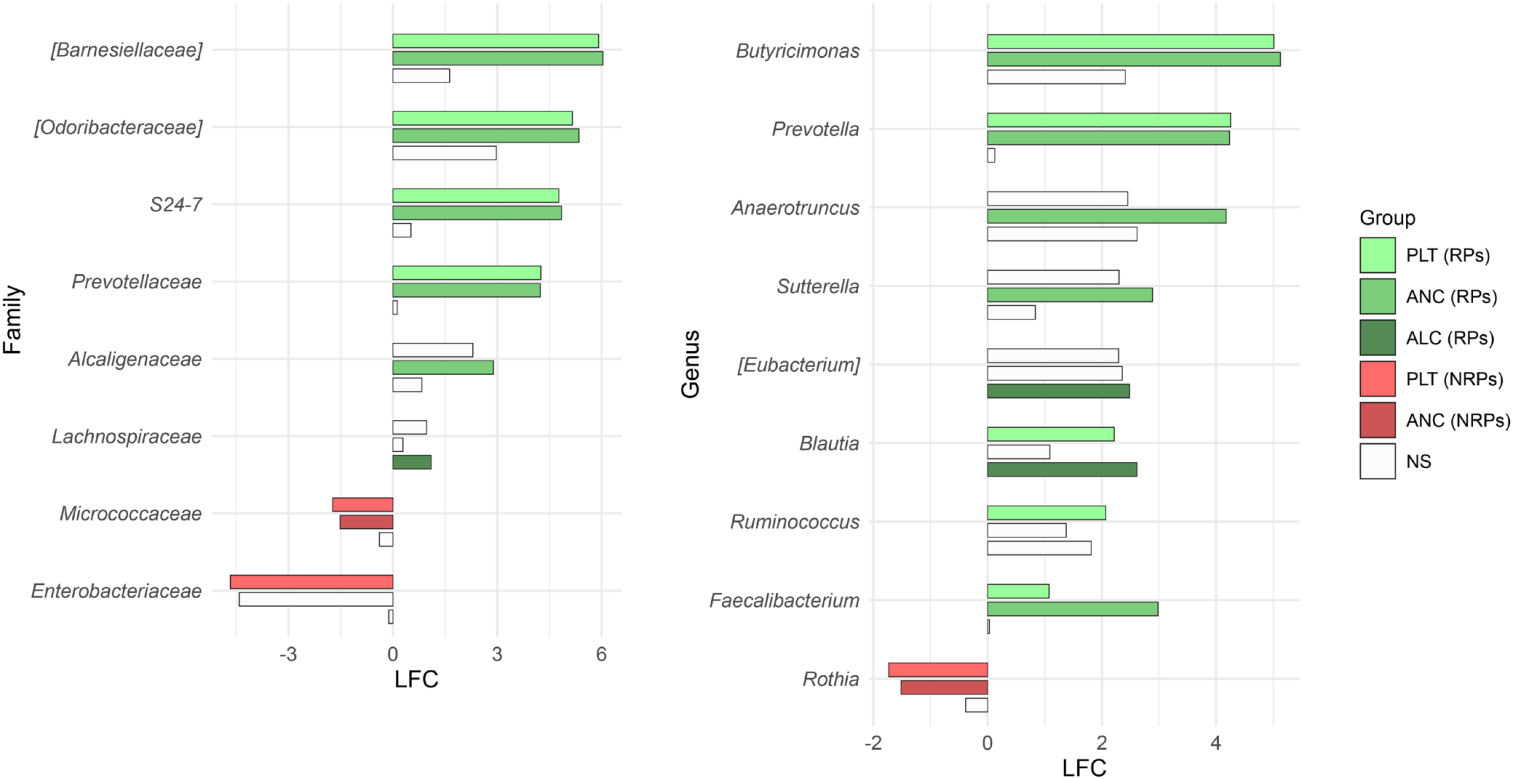
Gut microbiota signatures of hematologic recovery in AML patients at diagnosis. Bar plots showing log fold change (LFC) in the relative abundance distribution of families (left) and genera (right) that were differentially represented between patients who recovered (RPs) platelet count (PLT), absolute lymphocyte count (ALC), or absolute neutrophil count (ANC) and those who did not recover (NRPs) on Day 28 after induction therapy. LFC = $\log\left(\frac{c_{1}\cdot {\text{Median}}_{1}+\left(1-c_{1}\right)\cdot {\text{Mean}}_{1}+0.001}{c_{2}\cdot {\text{Median}}_{2}+\left(1-c_{2}\right)\cdot {\text{Mean}}_{2}+0.001}\right)$ where *c*1 = *c*2 = 0.7. Wilcoxon's test was used to identify significantly different taxa, and only taxa with FDR‐corrected *p*‐values < 0.1 are shown. Only taxa with a prevalence greater than 0.2 in at least two samples were included, resulting in 34 families and 42 genera tested. Bars are colored by group that showed significant values: Green shades indicate RPs for PLT, ANC, and ALC; red shades indicate NRPs for PLT and ANC; and white ones indicates taxa with no significant difference (NS). *N* = 22.

**FIGURE 6:**
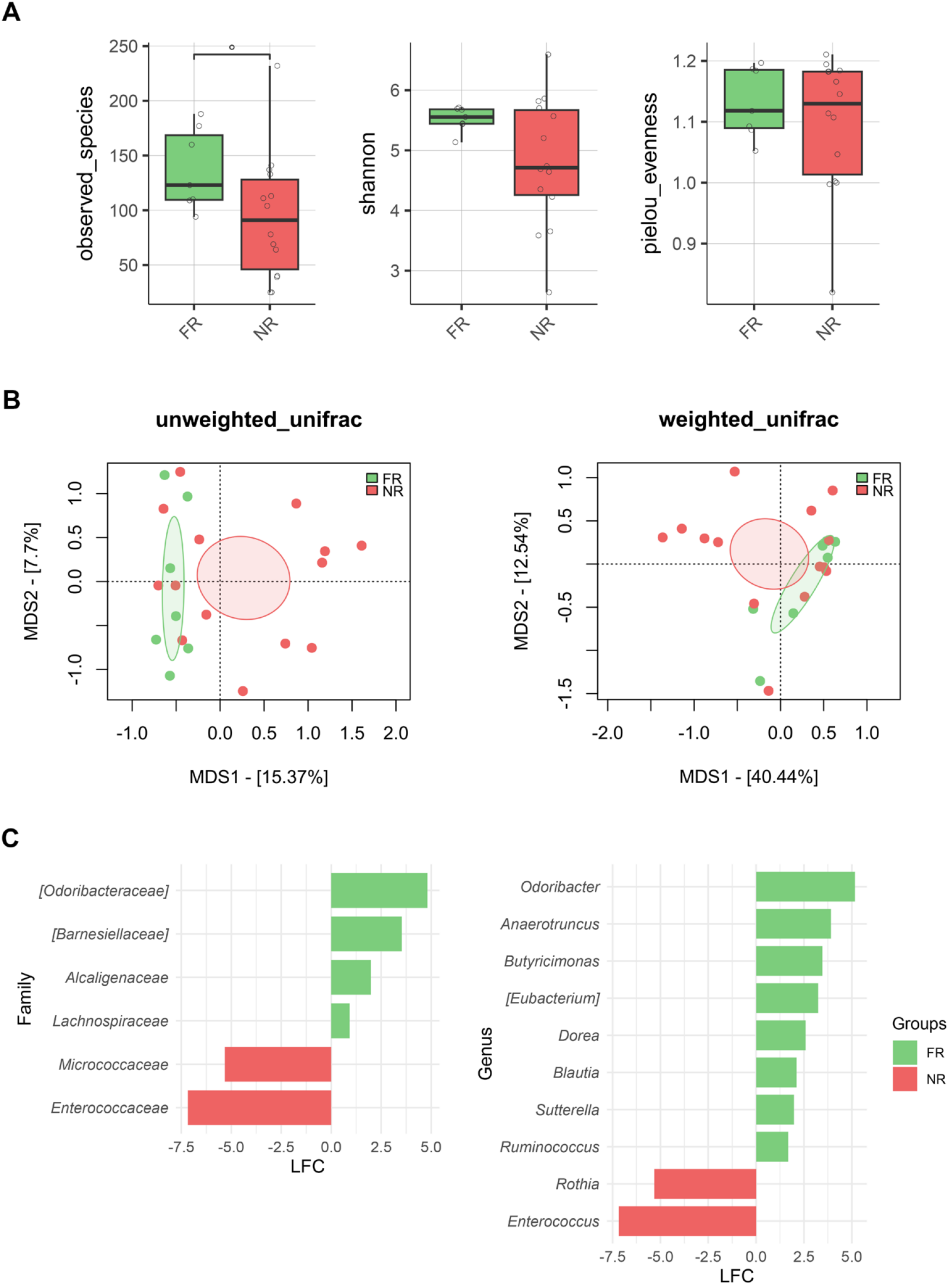
Gut microbiota diversity and taxonomic signatures associated with hematological recovery in AML patients at diagnosis. (A) Alpha diversity comparisons between patients with full hematological recovery (FR) and those who did not recover (NR) by Day 28 post‐induction therapy, represented by boxplots for three metrics: Observed species, Shannon index, and Pielou's evenness. Differences were assessed using the Wilcoxon test, with° indicating *p* < 0.1. (B) PCoAs illustrating beta diversity differences between FR and NR groups based on unweighted and weighted UniFrac distances. PERMANOVA was used to evaluate differences in community composition. (C) Bar plots showing log fold change (LFC) in the relative abundance of families (left) and genera (right) that were differentially represented between FR and NR patients. Wilcoxon's test was used to identify significantly different taxa, with FDR‐corrected *p*‐values < 0.1. Only taxa with a prevalence greater than 0.2 in at least two samples were included, resulting in 34 families and 42 genera tested. *N* = 22.

**FIGURE 7:**
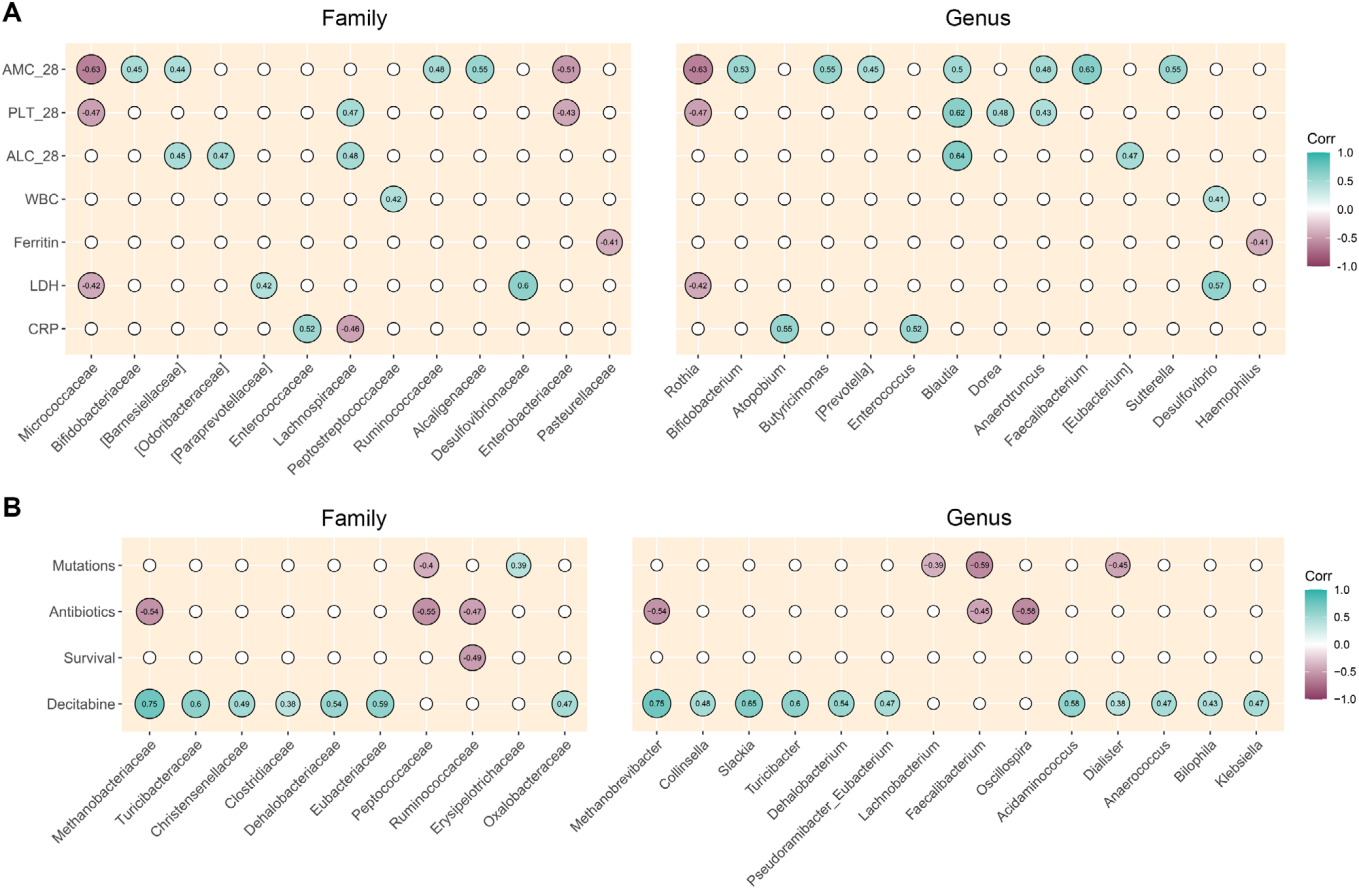
Correlations between relative taxon abundances and patient metadata. (A) Correlation plot showing Spearman's correlations between relative abundances at family or genus level and hematologic parameters. ALC_28, absolute lymphocyte count on Day 28 after therapy; ANC_28, absolute neutrophil count on Day 28 after therapy; CRP, C‐reactive protein; LDH, lactate dehydrogenase; PLT_28, platelet count on Day 28 after therapy; WBC, white blood cells. (B) Correlation plot showing point‐biserial correlation values between relative abundances at family or genus level and dichotomous patient metadata, i.e., oncogene mutations, antibiotic therapy, overall survival, and decitabine treatment. Only significant (*p* < 0.05) correlations with absolute rho (for Spearman correlations) or rpb (for point‐biserial correlations) > 0.3 are shown. Only taxa with a prevalence greater than 0.2 in at least two samples were included, resulting in 34 families and 42 genera tested.

We found an association between GM diversity and composition at diagnosis and hematologic recovery (particularly PLT and ANC) at Day 28 after therapy, which emerged as the most robust time point for evaluation. Earlier time points (Days 15 and 21) showed weaker associations and limited variability in hematological parameters, such as ANC, while Day 28 provided more significant and informative associations, making it the most suitable for analysis. In particular, patients who recovered PLT (RPs) had higher baseline alpha diversity than those who did not (NRPs) (Wilcoxon's test, *p* = 0.002) (Figure 3A). Moreover, PCoA based on both unweighted and weighted UniFrac distances showed a significant segregation between PLT RPs and NRPs (PERMANOVA, p 0.02) (Figure 3B). Similar results were obtained for alpha (Wilcoxon's test, *p* = 0.05) and beta diversity (PERMANOVA, unweighted UniFrac, *p* = 0.04; weighted UniFrac, *p* = 0.1) of patients stratified by ANC recovery (Figure 3C,D), while no differences were found when considering ALC recovery (*p* = 0.2) (data not shown). GM alpha diversity was also positively correlated with both PLT and ANC recovery (point‐biserial correlation rpb ≥ 0.47; *p* ≤ 0.03) (Figure 4). Taxonomically, PLT RPs showed increased proportions of the families [*Barnesiellaceae*], *Prevotellaceae*, S24‐7 and [*Odoribacteraceae*], and the genera *Ruminococcus* (from *Ruminococcaceae*), *Blautia*, *Faecalibacterium*, *Butyricimonas*, and *Prevotella* compared to NRPs (Wilcoxon's test with FDR correction, *p* < 0.05) (Figure 5 and Figure S6). On the other hand, PLT NRP patients were enriched in *Micrococcaceae*, including *Rothia* and *Enterobacteriaceae* (*p* ≤ 0.05). Partially overlapping results were obtained for ANC RPs, who were enriched in the families [*Barnesiellaceae*], S24‐7, [*Odoribacteraceae*] and *Alcaligenaceae*, and the genera *Faecalibacterium*, *Butyricimonas*, *Sutterella*, and *Anaerotruncus* compared to ANC NRPs (*p* < 0.05). ANC RPs also displayed trends similar to PLT RPs, including an increase in *Prevotellaceae* and *Prevotella* and a decrease in *Micrococcaceae* relative abundance compared to ANC NRPs (*p* ≤ 0.06). *Lachnospiraceae* and *Blautia*, together with [*Eubacterium*], were more represented in the baseline GM profile of ALC RPs compared to their NRP counterparts (*p* ≤ 0.03). The above GM signatures were generally confirmed when considering integrated hematologic recovery (i.e., PLT, ANC, and ALC together) (Figure 7). Briefly, patients who fully recovered (FRs) tended to have higher alpha diversity than those who did not (NRs) (observed species, Wilcoxon's test with FDR correction, *p* = 0.079) and were significantly segregated in the unweighted UniFrac‐based PCoA (PERMANOVA, *p* = 0.021). These diversity results suggest that differences in hematologic recovery may be more strongly associated with the presence or absence of specific taxa, rather than the relative abundances of taxa present. From a taxonomic perspective (Figure 6C and Figure S7) at the family level, FR showed increased abundances of [*Odoribacteraceae*], [*Barnesiellaceae*], and *Alcaligenaceae*, along with a reduced presence of *Micrococcaceae* and *Enterococcaceae*. At the genus level, FR were characterized by higher proportions of *Blautia*, *Butyricimonas*, *Eubacterium*, *Odoribacter*, *Anaerotruncus*, *Dorea*, *Sutterella*, and *Ruminococcus*, and lower proportions of *Rothia* and *Enterococcus* (*p* < 0.1).

### 3.5 Correlations Between Baseline Relative Taxon Abundances and Patient Metadata

Finally, we sought correlations between relative taxon abundances at diagnosis and hematologic variables (Figure 7A), as well as other patient metadata (Figure 7B). PLT on Day 28 correlated positively with *Lachnospiraceae*, *Blautia*, *Dorea*, and *Anaerotruncus* (Spearman's rho ≥ 0.43; *p* ≤ 0.05), and negatively with *Micrococcaceae*, *Enterobacteriaceae*, and *Rothia* (rho ≤ −0.43; *p* ≤ 0.05). ALC on Day 28 correlated directly with *[Barnesiellaceae]*, *[Odoribacteraceae]*, *Lachnospiraceae*, *Blautia*, and *[Eubacterium]* (rho ≥ 0.04; *p* ≤ 0.03). Finally, ANC on Day 28 correlated positively with *Bifidobacteriaceae*, *[Barnesiellaceae]*, *Ruminococcaceae*, *Alcaligenaceae*, *Bifidobacterium*, *Butyricimonas*, *[Prevotella]*, *Blautia*, *Anaerotruncus*, *Faecalibacterium*, and *Sutterella* (rho ≥ 0.44; *p* ≤ 0.04), and negatively with *Micrococcaceae*, *Enterobacteriaceae*, and *Rothia* (rho ≤ −0.43; *p* ≤ 0.02). Regarding other hematologic parameters at baseline, we found a positive correlation between WBCs and *Peptostreptococcaceae* and *Desulfovibrio* (rho ≥ 0.42; *p* ≤ 0.04); a negative correlation between ferritin and *Pasteurellaceae* and *Haemophilus* (rho = −0.4; *p* = 0.04); a positive correlation between lactate dehydrogenase and *[Paraprevotellaceae]*, *Desulfovibrionaceae*, and *Desulfovibrio* (rho ≥ 0.42; *p* ≤ 0.03), and negative with *Micrococcaceae* and *Rothia* (rho = −0.42; *p* = 0.03); and a positive correlation between CRP and *Enterococcaceae*, *Atopobium*, and *Enterococcus* (rho: ≥ 0.52; *p* ≤ 0.004), while negative with *Lachnospiraceae* (rho = −0.46; *p* = 0.02). For dichotomous variables, the presence of pro‐oncogenic mutations correlated positively with *Erysipelotrichaceae* (point‐biserial correlation rpb = 0.39; *p* = 0.045) and negatively with *Peptococcaceae*, *Lachnobacterium*, *Faecalibacterium*, and *Dialister* (rpb ≤ −0.39; *p* ≤ 0.05). Antibiotic use correlated negatively with *Methanobacteriaceae*, *Peptococcaceae*, *Ruminococcaceae*, *Methanobrevibacter*, and *Oscillospira* (rpb ≤ −0.45; *p* ≤ 0.02). OS correlated inversely with *Ruminococcaceae* (rpb = −0.49; *p* = 0.01). The use of hypomethylating agent decitabine was positively correlated with many different taxa, such as *Methanobacteriaceae*, *Turicibacteraceae*, *Christensenellaceae*, *Clostridiaceae*, *Dehalobacteriaceae*, *Eubacteriaceae*, *Oxalobacteraceae*, *Methanobrevibacter*, *Collinsella*, *Slackia*, *Turicibacter*, *Dehalobacterium*, *Pseudoramibacter*_*Eubacterium*, *Acidaminococcus*, *Dialister*, *Anaerococcus*, *Bilophila*, and *Klebsiella* (rpb ≥ 0.38; *p* < 0.05).

## 4 Discussion

As far as we know, this is the first study to identify potential baseline gut microbiome (GM) signatures associated with hematological recovery after induction therapy in AML patients, supporting the already known role of GM in hematopoietic reconstitution [31].

Recent studies have shown that GM members can influence hematopoiesis by actively controlling hematopoietic stem cell precursors and supporting myelopoiesis and lymphopoiesis, ultimately contributing to enhanced resistance to infection in mice [31]. Supporting this evidence, GM dysbiosis has been linked to reduced hematopoiesis in both humans and mice [32, 33]. Recent research in mice also suggests that antibiotic‐induced depletion of some GM members and subsequent suppression of bone marrow function are associated with the absence of heat‐stable microbial products [32]. These products, when present, circulate in the bloodstream and stimulate hematopoiesis by initiating basal inflammatory signaling. Therefore, it is not surprising that in our study cohort, a better hematologic recovery following chemotherapy is associated with a healthy‐like GM composition. In particular, patients who recovered PLT, ALC, and ANC at 28 days after therapy differed from their NRP counterparts on several GM features even before treatment was initiated. Such features included higher alpha diversity, a recognized hallmark of GM eubiosis, in PLT and ANC RPs, as well as several compositional differences between RPs and NRPs. Notably, all RPs were enriched in health‐associated taxa, including *Ruminococcus, Blautia, Butyricimonas*, and *Faecalibacterium*. These genera are capable of producing short‐chain fatty acids (SCFAs), mainly butyrate, which play a key role in maintaining host health, including metabolic, immunological, and neurological homeostasis [34]. In particular, SCFAs have been shown to modulate hematopoiesis, fostering the generation of hematopoietic precursor cells, the differentiation of various immune system cells, and the resultant anti‐cancer effects [33, 35, 36], and specifically to enhance adoptive immunotherapy of cancer through metabolic and epigenetic reprogramming [37]. Furthermore, gavage with butyrate or one of its main producers, *Faecalibacterium*, has been shown to delay murine AML progression, probably by repairing intestinal barrier damage and inhibiting lipopolysaccharide absorption and leakage into the blood [15]. Therefore, the presence of these beneficial taxa (and their metabolites) at diagnosis may contribute in various ways to improved hematologic recovery of AML patients after induction therapy. It should be noted that *Faecalibacterium* was underrepresented in patients on antibiotic therapy prior to sample collection, who also showed reduced alpha diversity, highlighting the need to carefully reconsider antibiotic stewardship programs to avoid adverse GM‐related outcomes, as recently discussed [38, 39]. ANC RPs exhibited an overabundance of *Sutterella*, a taxon with a complex role in human physiology. While *Sutterella* has been linked to conditions like inflammatory bowel disease and autism spectrum disorders [40], it also supports gut barrier integrity, potentially reducing systemic inflammation. Notably, *Sutterella* has been associated with positive outcomes, such as complete remission and prolonged survival following CAR‐T cell therapy for hematologic malignancies. This suggests that *Sutterella* may play a supportive role in immune modulation and recovery, aligning with its enrichment in ANC RPs in our study [41]. Finally, as expected, PLT RPs were depleted in potentially pathogenic taxa, particularly *Enterobacteriaceae* and *Micrococcaceae*, including *Rothia*, whose infections, although rare, have been shown to cause significant morbidity and mortality in immunocompromised hosts, including AML patients [42, 43].

Some study limitations should be mentioned, including the small sample size, the associative nature of the study, and the broad‐spectrum antibiotic therapy that approximately half of the patients received prior to sample collection, which may have partially biased our data. However, it should be noted that compositional differences between patients receiving antibiotics and those not receiving antibiotics were mainly accounted for by taxa other than those related to hematologic recovery. Among these, the enrichment in *Lactococcus*, also shared by those who developed post‐therapy infections, merits further investigation. An additional limitation is the lack of longitudinal sampling to reconstruct changes over time. Induction therapy is known to cause profound disruptions to the GM, characterized by a loss of microbial diversity, depletion of beneficial taxa such as *Faecalibacterium* and *Bifidobacterium*, and the enrichment of opportunistic pathogens like *Enterococcus* [17]. These alterations, as reported by Rashidi et al., often persist long after treatment, resulting in a restructured microbiota [44]. Despite these changes, baseline GM composition has emerged as a reliable predictor of clinical outcomes, including in various cancer contexts [45, 46]. Higher baseline microbiome diversity has been linked to a reduced infection risk during neutropenia in AML patients [10]. Similarly, identified protective baseline GM profiles have been associated with a lower incidence of major infections in patients undergoing HSCT [47]. These findings highlight the importance of the initial GM, even in the context of significant post‐treatment dysbiosis.

Although further studies in larger cohorts are needed to validate our findings, along with animal models for mechanistic insights (also in relation to the seemingly weaker link between GM and ALC), this pilot study highlighted early GM features that may be a potential marker of hematologic recovery in AML patients undergoing induction therapy. Assessment of the pretreatment GM profile could thus play a pivotal role in personalized risk stratification and the design of precision GM‐based interventions to optimize recovery in AML patients undergoing induction therapy and potentially improve overall outcomes in individuals with hematologic diseases.

## Author Contributions

**Valentina Salvestrini:** conceptualization (lead), project administration (lead), writing – original draft (equal). **Gabriele Conti:** data curation (equal), formal analysis (lead), methodology (equal), writing – original draft (equal). **Federica D'Amico:** formal analysis (equal), methodology (equal), writing – original draft (equal). **Gianluca Cristiano:** data curation (equal), writing – original draft (equal). **Marco Candela:** writing – review and editing (supporting). **Michele Cavo:** writing – review and editing (supporting). **Silvia Turroni:** funding acquisition (equal), writing – review and editing (supporting). **Antonio Curti:** funding acquisition (equal), writing – review and editing (supporting).

## Ethics Statement

The study was conducted in accordance with the Declaration of Helsinki and approved by the Independent Ethics Committee of the Area Vasta Emilia Centro (approval code: 94/2016/O/Tess).

## Consent

Written informed consent was obtained from all patients prior to their participation in the study.

## Conflicts of Interest

The authors declare no conflicts of interest.

## Supporting information


Data S1.

## Data Availability

The data supporting the findings of this study are available in the NCBI Sequence Read Archive (SRA) under BioProject accession number PRJNA1139414.
