# Supplementary material for: Gut Microbiome as a Potential Marker of Hematologic Recovery Following Induction Therapy in Acute Myeloid Leukemia Patients
Source: Cancer Med. 2025 Jan 27;14(3):e70501. doi: 10.1002/cam4.70501 (PMC11770270; doi:10.1002/cam4.70501)
Supplement: Supplementary file 1 — Data S1. [file CAM4-14-e70501-s001.docx]

**Supplementary Table S1 Antibiotic treatments given within 30 days prior to sampling**

| **#Pts** | Antibiotics | Duration  (days) | Neutropenia prophylaxis | Duration  (days) |
| --- | --- | --- | --- | --- |
| **1** |  |  | Levofloxacin | 3 |
| **2** |  |  | Levofloxacin | 4 |
| **3** |  |  | Levofloxacin | 6 |
| **4** |  |  | Levofloxacin | 3 |
| **5** | Ceftazidime | 3 | Levofloxacin | 4 |
| **6** |  |  |  |  |
| **7** |  |  |  |  |
| **8** |  |  |  |  |
| **9** | Amoxicillin | 6 | Levofloxacin | 4 |
| **10** |  |  |  |  |
| **11** | Tigecycline | 3 | Levofloxacin | 9 |
| **12** |  |  |  |  |
| **13** |  |  | Levofloxacin | 5 |
| **14** |  |  | Levofloxacin | 7 |
| **15** |  |  |  |  |
| **16** | Amoxicillin | 3 |  |  |
| **17** |  |  |  |  |
| **18** | Piperacillin/Tazobactam | 5 |  |  |
| **19** |  |  |  |  |
| **20** | Amikacin, Piperacillin/Tazobactam | 1 |  |  |
| **21** |  |  | Levofloxacin | 24 |
| **22** |  |  |  |  |
| **23** | Piperacillin/Tazobactam | 4 |  |  |
| **24** |  |  |  |  |
| **25** | Piperacillin/Tazobactam | 10 | Levofloxacin | 5 |
| **26** | Meropenem, Linezolid | 8 | Levofloxacin | 18 |
| **27** |  |  | Levofloxacin | 2 |

**Supplementary Figures**

**Figure S1. Hematologic recovery in AML patients after induction therapy**

Hematologic recovery was assessed at days +15, +21, +28 in AML patients after induction therapy, and immediately before consolidation therapy. (A) Absolute lymphocyte count (ALC, cells/mm³). (B) Absolute neutrophil count (ANC, cells/mm³). (C) Platelet count (PLT, cells/mm³). Each line represents the recovery trajectory of an individual patient (AML_FC_x), illustrating inter-patient variability in hematologic recovery patterns

**
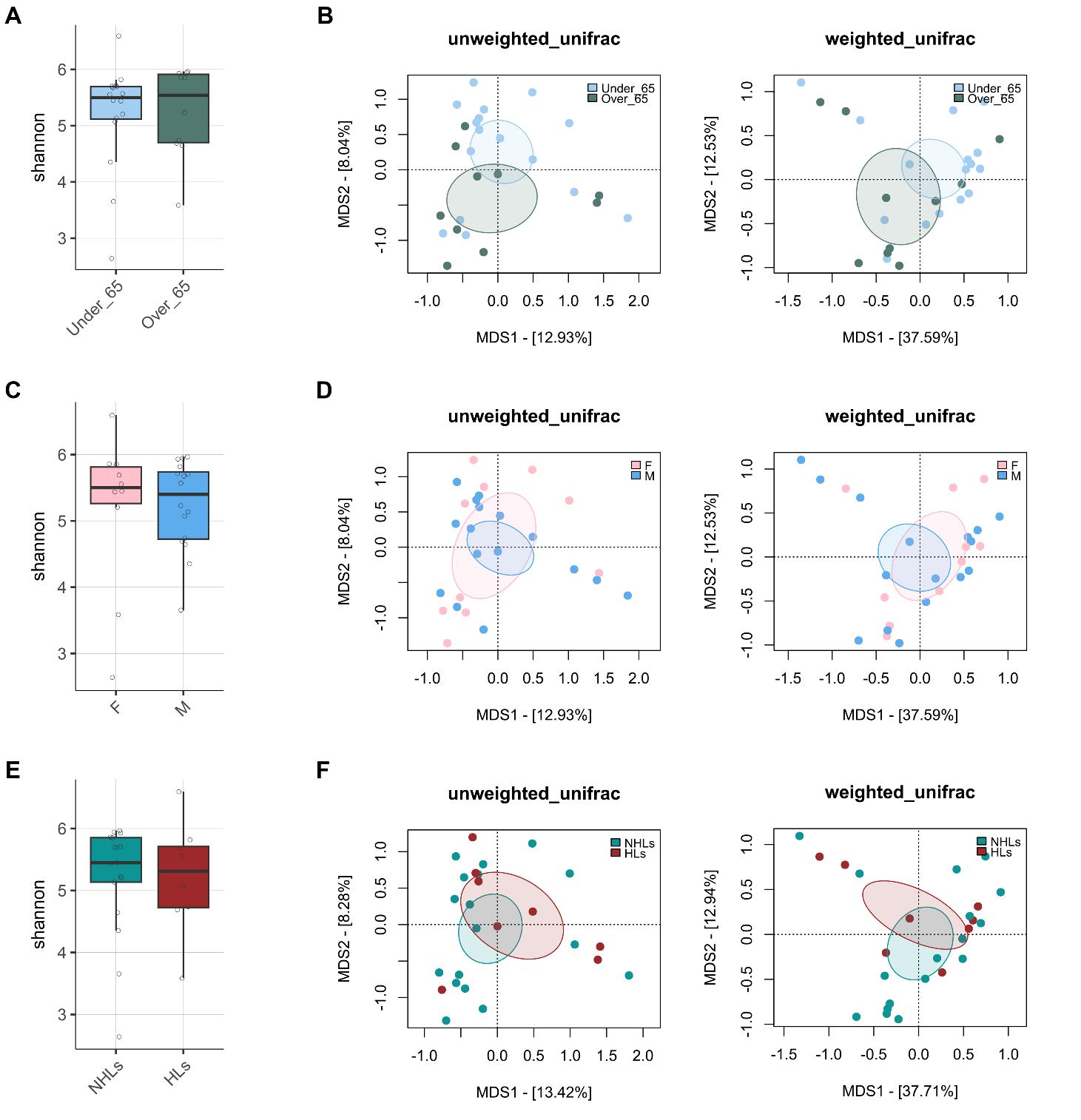
Figure** **S2. Association of alpha and beta diversity with age, sex, and WBC.**

Alpha diversity was assessed using the Shannon index, which accounts for both richness and evenness of microbial communities, while beta diversity was evaluated using unweighted and weighted UniFrac distances to capture compositional dissimilarity based on phylogenetic relationships. For alpha diversity comparisons, the Wilcoxon rank-sum test with FDR p-value correction was applied, and for beta diversity, PERMANOVA was used to test for differences between groups. Ellipses include 95% confidence intervals based on the standard error of the weighted average of sample coordinates. No significant separation was found (PERMANOVA, *p* > 0.1) (A-B) Association of alpha and beta diversity with age (n° = 26), with patients stratified into "Under 65" and "Over 65" groups. (C-D) Association of alpha and beta diversity with sex (n = 26), categorized as "M" (male) and "F" (female). (E-F) Association of alpha and beta diversity with WBC levels (n = 25), with groups "NHLs" (Not-hyperleukocytosis) and "HLs" (Hyperleukocytosis).

**
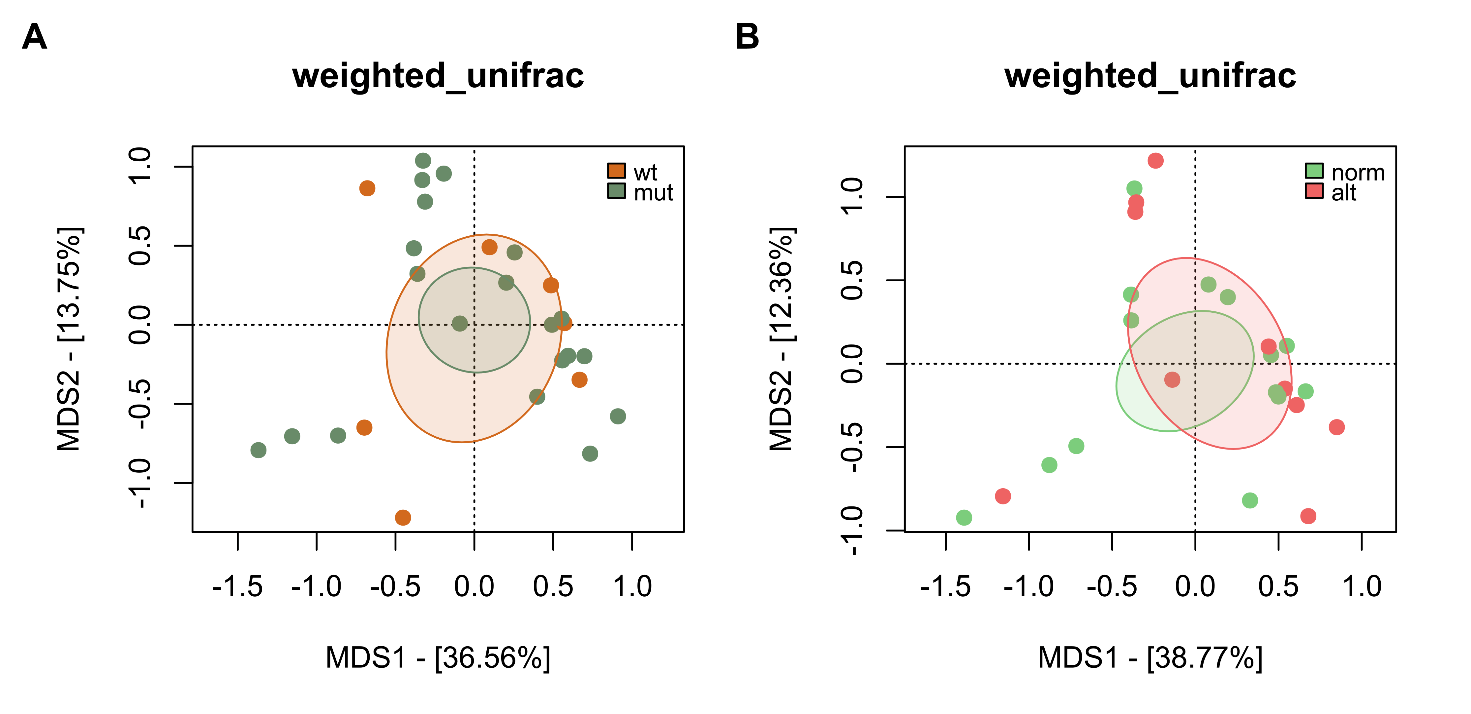
**

**Figure S3. Beta diversity of the gut microbiota of AML patients at diagnosis stratified by oncogene mutations and karyotype alterations.** PCoA plots based on weighted UniFrac distances between gut microbiota profiles of patients stratified, presence ("mut") or absence ("wt") of mutations in *FLT3*, *NPM1, IDH1/2,* and *TP53* genes (**A**), or karyotype alterations ("alt") compared to "norm" (46 xx, 46 xy; **B**). Ellipses include 95% confidence intervals based on the standard error of the weighted average of sample coordinates. No significant separation between study groups was found (PERMANOVA, *p >* 0.1).


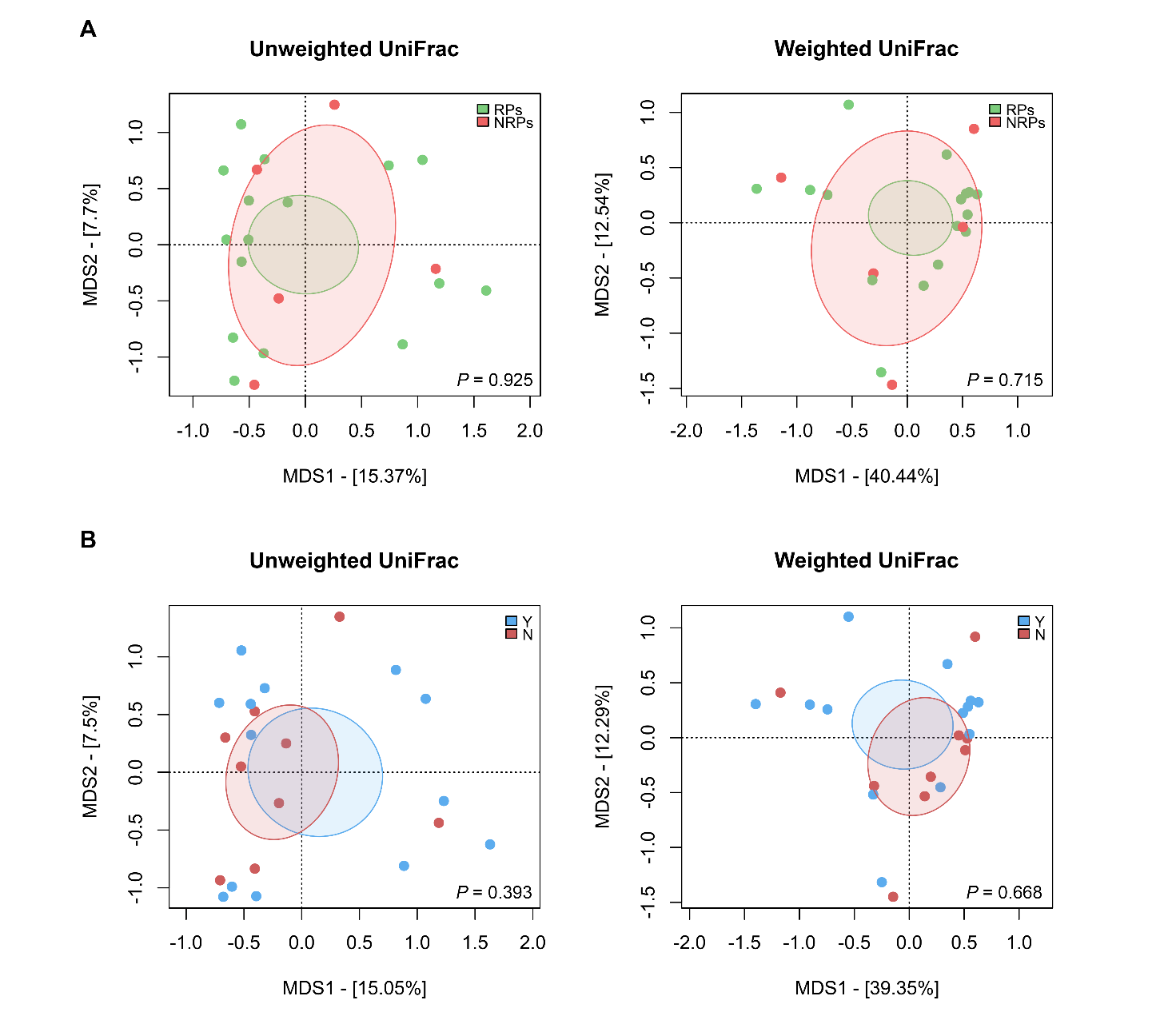


**Figure S4. Beta diversity of the gut microbiota of AML patients at diagnosis stratified by response to therapy (excluding those treated with decitabine).** PCoA plots based on unweighted and weighted UniFrac distances between gut microbiota profiles of patients who responded (RPs) or did not respond (NRPs) to induction therapy (**A**) and between patients stratified by OS (**B**), who survive (Y) or did not survive (N). Patients treated with decitabine were excluded. Ellipses include 95% confidence intervals based on the standard error of the weighted average of sample coordinates. No significant separation was found (PERMANOVA, *p* > 0.39).

**
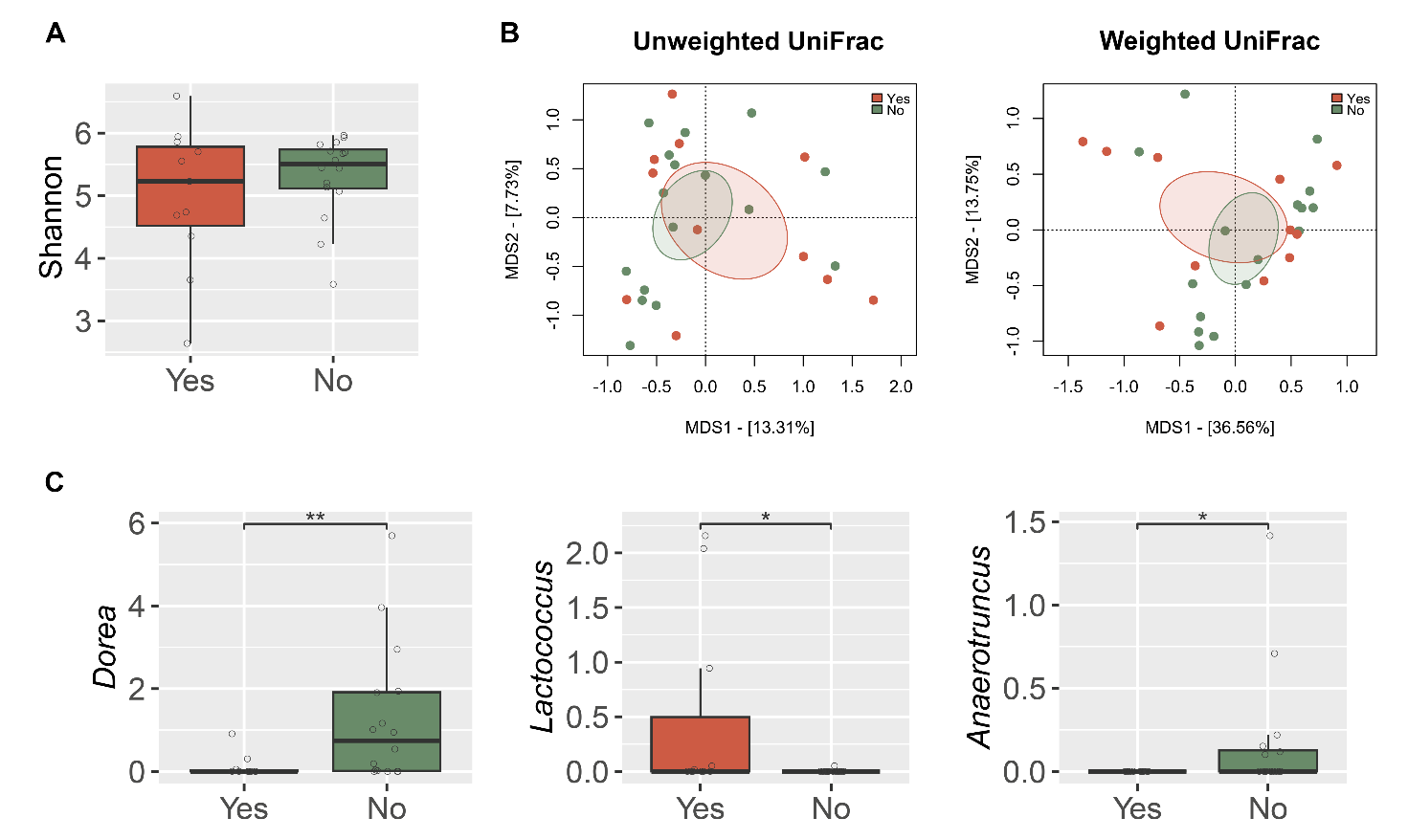

Figure S5. The gut microbiota of AML patients at diagnosis stratified by the occurrence of post-therapy infections.** (**A**) Boxplot showing the distribution of alpha diversity, estimated with the Shannon index, in the gut microbiota of patients who developed a post-therapy infection (Yes) or not (No). Wilcoxon test, *p* = 0.68. (**B**) PCoA plots based on unweighted and weighted UniFrac distances between gut microbiota profiles. Ellipses include 95% confidence intervals based on the standard error of the weighted average of sample coordinates. A trend towards segregation was found in the weighted UniFrac-based PCoA (PERMANOVA, *p* = 0.089). (**C**) Boxplots showing the relative abundance distribution of genera differentially represented between groups. Wilcoxon test, * for *p* < 0.05, ** for *p* < 0.01.


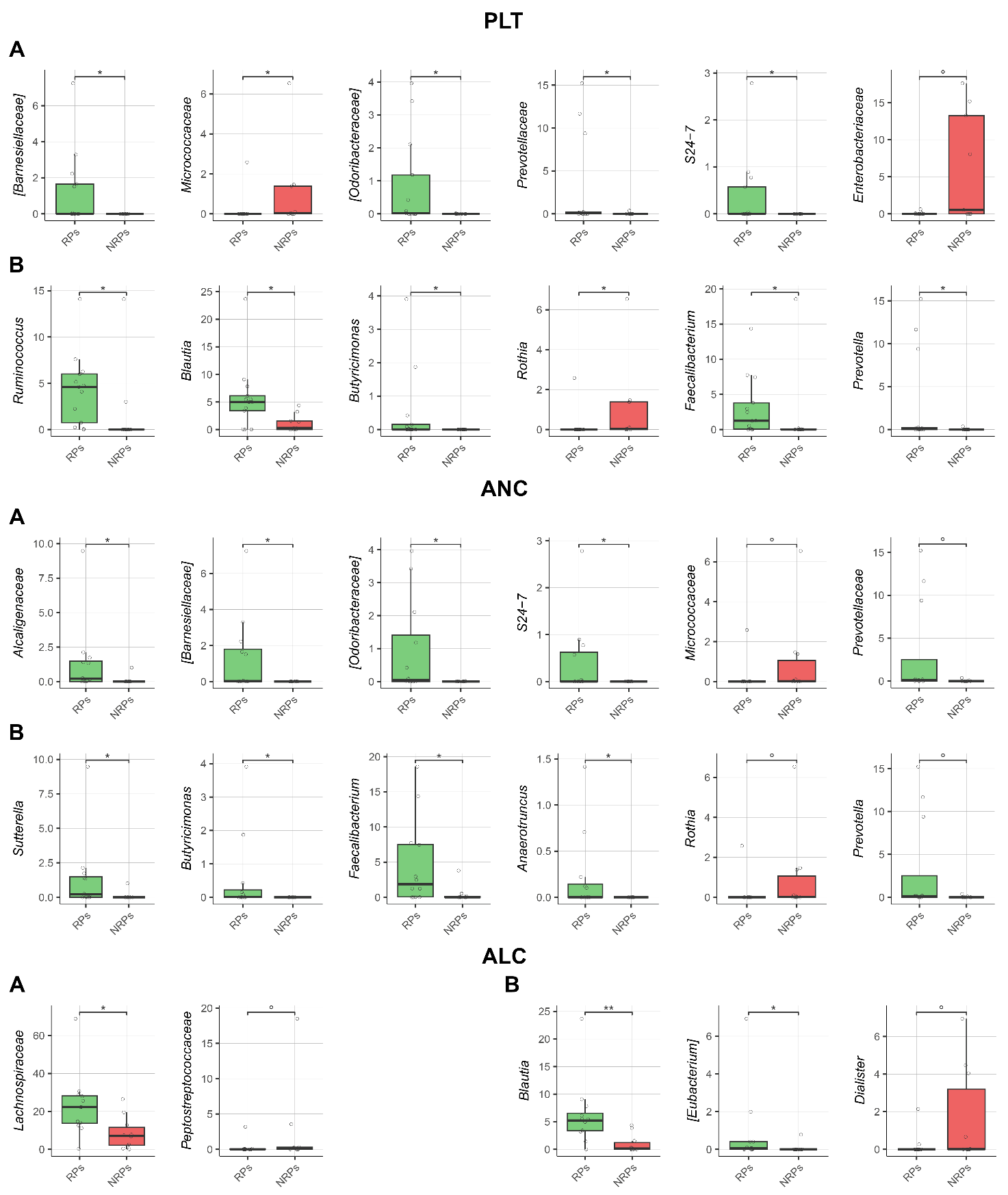


**Figure S6. Taxonomic signatures of gut microbiota associated with hematological recovery on day 28 in AML patients at diagnosis.**

Boxplots showing the relative abundance distribution of families (**A**) and genera (**B**) differentially represented between patients who recovered platelet count (PLT 28), absolute lymphocyte count (ALC 28) or absolute neutrophil count (ANC 28) (RPs) and those who did not (NRPs) on day 28 after induction therapy. Wilcoxon test with FDR-corrected *p*-value, ° *p* < 0.1, * *p* < 0.05, ** *p* < 0.01. N = 22.
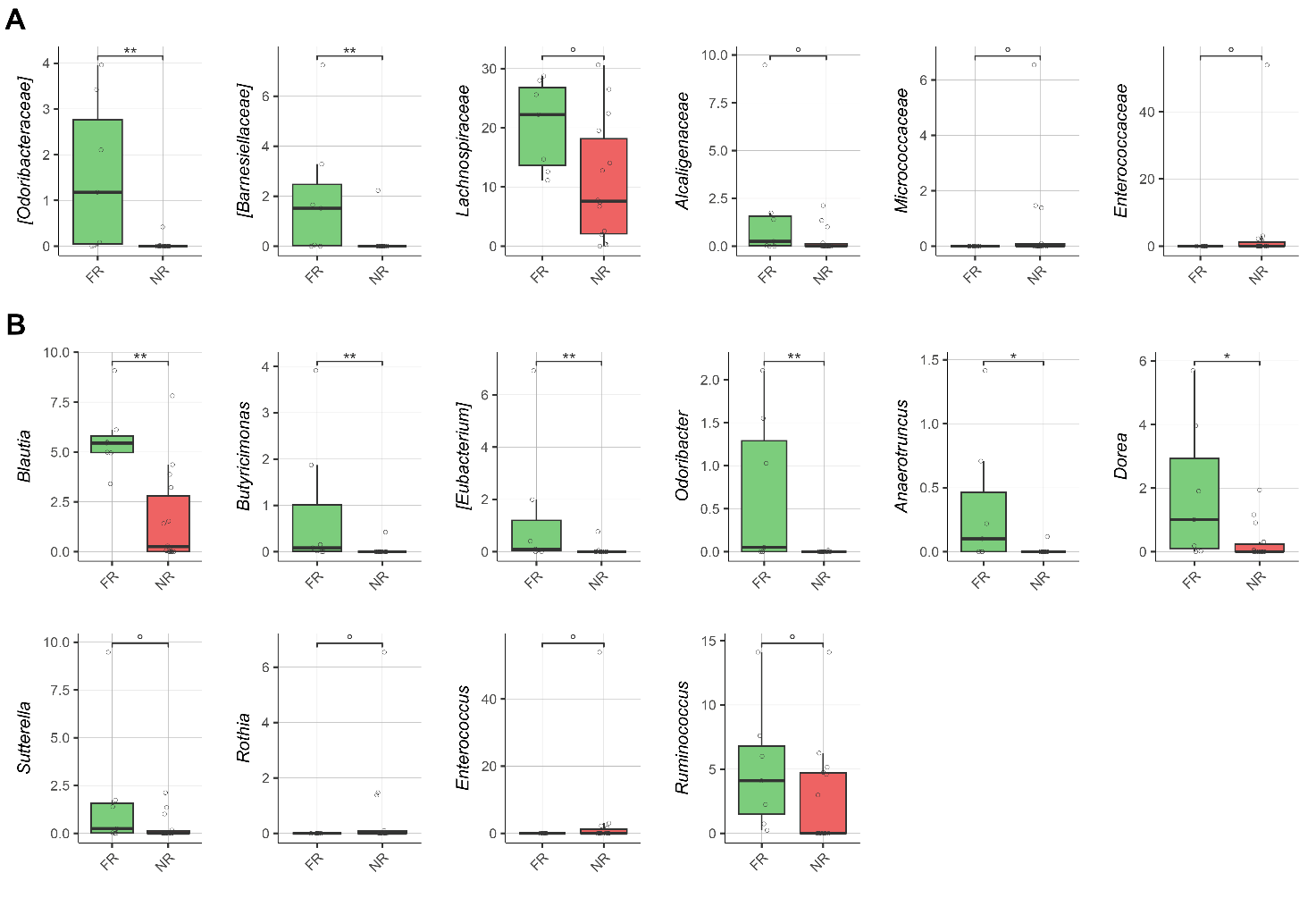


**Figure S7. Taxonomic signatures of gut microbiota associated with full hematological recovery on day 28 in AML patients at diagnosis.**

Boxplots showing the relative abundance distribution of families (**A**) and genera (**B**) differentially represented between patients with full hematological recovery (FR) and those who did not recover (NR) by day 28 post-induction therapy. Wilcoxon test with FDR-corrected *p*-value, ° *p* < 0.1, * *p* ≤ 0.05, ** *p* < 0.01. N = 22.
